# Cardiac specific knock-down of peroxisome proliferator activated receptor α prevents fasting-induced cardiac lipid accumulation and reduces perilipin 2

**DOI:** 10.1371/journal.pone.0265007

**Published:** 2022-03-08

**Authors:** Natasha Fillmore, Vincent Hou, Junhui Sun, Danielle Springer, Elizabeth Murphy

**Affiliations:** 1 Laboratory of Cardiac Physiology, National Heart, Lung and Blood Institute, National Institutes of Health, Bethesda, Maryland, United States of America; 2 Department of Pharmaceutical Sciences, North Dakota State University, Fargo, North Dakota, United States of America; 3 Murine Phenotyping Core, National Heart, Lung and Blood Institute, National Institutes of Health, Bethesda, Maryland, United States of America; UAB School of Medicine, UNITED STATES

## Abstract

While fatty acid metabolism is altered under physiological conditions, alterations can also be maladaptive in diseases such as diabetes and heart failure. Peroxisome Proliferator Activated Receptor α (PPARα) is a transcription factor that regulates fat metabolism but its role in regulating lipid storage in the heart is unclear. The aim of this study is to improve our understanding of how cardiac PPARα regulates cardiac health and lipid accumulation. To study the role of cardiac PPARα, tamoxifen inducible cardiac-specific PPARα knockout mouse (cPPAR^-/-^) were treated for 5 days with tamoxifen and then studied after 1–2 months. Under baseline conditions, cPPAR^-/-^ mice appear healthy with normal body weight and mortality is not altered. Importantly, cardiac hypertrophy or reduced cardiac function was also not observed at baseline. Mice were fasted to elevate circulating fatty acids and induce cardiac lipid accumulation. After fasting, cPPAR^-/-^ mice had dramatically lower cardiac triglyceride levels than control mice. Interestingly, cPPAR^-/-^ hearts also had reduced Plin2, a key protein involved in lipid accumulation and lipid droplet regulation, which may contribute to the reduction in cardiac lipid accumulation. Overall, this suggests that a decline in cardiac PPARα may blunt cardiac lipid accumulation by decreasing Plin2 and that independent of differences in systemic metabolism a decline in cardiac PPARα does not seem to drive pathological changes in the heart.

## Introduction

Fatty acid metabolism plays an important physiological role in the heart where fatty acid oxidation typically supplies the majority of ATP [1]. However, dysregulation of fat metabolism can have dramatic deleterious effects. Elevations in fatty acid oxidation have been implicated in driving cardiac insulin resistance and increasing the risk of developing cardiac dysfunction and heart failure [2–8]. On the other hand, in severe heart failure a decline in overall mitochondrial oxidative metabolism (including fatty acid oxidation) has detrimental effects on cardiac function [7, 9–13]. Thus fatty acid metabolism in the heart needs to be carefully regulated.

Free fatty acids enter the heart via fatty acid transporters were they are converted to triacylglycerol (TAG) and stored in lipid droplets (LD). In the heart, most fatty acids used for β-oxidation are generated from LD. LD have a core of neutral lipids enveloped within a phospholipid membrane, which contains a number of proteins such as perilipins (Plin). Plin have been shown to shield the LD from lipases as well as regulate the activation of lipases adipose triglyceride lipase (ATGL) and hormone sensitive lipase (HSL) [14–20]. There are 5 members of the Plin family (Plin 1 to 5). Recent studies report an important role for Plin2 and Plin5, which are both highly expressed in heart, in regulating LD turnover in the heart [14–18]. Mice with cardiac specific overexpression of Plin2 [17] exhibit cardiac steatosis suggesting that Plin2 opposes breakdown of LD. Loss of Plin2 has also been reported to reduce lipophagy [18], a process in which LD are trafficked to the lysosome for degradation.

Peroxisome proliferator activated receptor (PPAR)α is a transcription factor that is an important regulator of whole body and cardiac fatty acid metabolism. PPARα is also reported to contribute to the development of cardiovascular diseases including cardiac hypertrophy, heart failure, and diabetic cardiomyopathy through its regulation of fat metabolism [8, 21–36]. Cardiac specific overexpression of PPARα elevates fatty acid oxidation and cardiac lipid accumulation, inducing cardiac dysfunction and increasing susceptibility to cardiac hypertrophy [2, 21, 22, 29, 34, 37]. Changes in binding partners of PPARα and changes in the activity of transcription factors/coactivators have been implicated in this role of PPARα in changes in gene expression and cardiac dysfunction and hypertrophy [8, 33, 38–40]. However, whole body PPARα knockout mice are also more susceptible to cardiac lipid accumulation [41–43] and there are inconsistent reports on the susceptibility of these mice to developing cardiac hypertrophy [8, 29, 30, 44]. The deleterious cardiac effects observed in the whole body PPARα knockout mice could be due to systemic effects of loss of PPARα in other tissues. PPARα elevates fatty acid oxidation by increasing the expression of many fatty acid metabolism enzymes including carnitine palmitoyl transferase (CPT) and CD36 [2, 21, 28, 32, 37, 45, 46]. In addition, PPARα has been shown to be a positive regulator of Plin [47–50], suggesting PPARα also regulates an additional aspect of fatty acid metabolism, lipid droplets.

To gain a better understanding of the role of cardiac PPARα in regulating cardiac metabolism we developed the first cardiac specific PPARα knockout mouse. This mouse is inducible and is deleted by addition of tamoxifen allowing us to study the role of PPARα in the adult heart. The aim of this study was to assess how myocardial PPARα regulates cardiac health and lipid accumulation during physiological stress, in particular increased fatty acid supply. In contrast to studies showing that a global reduction of PPARα can have a deleterious impact, we did not observe any dysfunction or cardiac hypertrophy in cPPAR^-/-^ mice at baseline. Interestingly, Plin2 was reduced in these cPPAR^-/-^ hearts. When we fasted these mice for 16 hours, we observed lower TAG levels in the cPPAR^-/-^ hearts along with a reduction in cardiac Plin2. Taken together, these data suggest that loss of cardiac PPARα decreases levels of Plin2 which may result in the reduced cardiac lipid accumulation.

## Methods

### Mice

Tamoxifen inducible cardiac-specific PPARα^-/-^ (cPPAR^-/-^) mice are on the C57BL6N background and are both floxed and express MerCreMer. The floxed PPARα mouse (PPAR^flox/flox^) was generated by breeding the PPARα First Knockout mouse (041228-UCD, received from Mutant Mouse Resource Research Center) with the Flp recombinase expressing mouse (036512-UCD, received from Mutant Mouse Resource Research Center). PPAR^flox/flox^ were then bred with αMHC MerCreMer mice (MerCreMer+/-) to produce an inducible cardiac specific PPARα Knockout mouse (MerCreMer+/-;PPAR^flox/flox^). MerCreMer+/- and PPAR^flox/flox^ results are together indicated as Control in the figures. To induce PPARα knockout, tamoxifen (20 mg/kg body weight each day; tamoxifen is dissolved in 5% ethanol and 95% peanut oil) was administered IP for 5 consecutive days. Echocardiography was performed on isoflurane anesthetized mice with a 30-MHz linear transducer and the Vevo 2100 system.

Mice were housed in an animal facility with a 12 hour light:12 hour dark cycle. Mice were provided standard chow and water *ad libitum* and at the start of each experiment were 3–4 months of age. In the fasted studies, mice were fasted overnight for 16 hr. Mouse treatment and handling followed guidelines outlined in the Guide for the Care and Use of Laboratory Animals (National Institutes of Health). All animal protocols were approved by the National Heart, Lung and Blood Institute’s Institutional Animal Care and Use Committee.

Mouse genotype (presence of MerCreMer and PPARα flox sites) was determined with PCR using these primer sets: MerCreMer, forward primer, 5’-GTCTGACTAGGTGTCCTTCT-3’ and reverse primer, 5’-CGTCCTCCTGCTGGTATAG-3’. These primers were used to amplify a 410 bp DNA fragment. Presence of PPARα flox forward primer, 5’-GTTCTTCCTGGGTATAGCCTTGACG-3’ and reverse primer, 5’-TGAGCCACAGCCCAGTCCTACC-3’. These primers were used to amplify a 454 bp DNA fragment if PPAR flox sites are present and a 277 bp DNA fragment if Wild Type. Finally, these primers were used to detect whether the Cre cut the flox sites (471 bp DNA fragment): forward primer, 5’-GTTCTTCCTGGGTATAGCCTTGACG-3’ and reverse primer, 5’-ATAGATGATTAAAAGGCTGATGTTAGGC-3’.

### Western blot

A standard protocol was followed to assess protein levels via western blot in mouse heart. Hearts that had been crushed in liquid nitrogen were homogenized in RIPA buffer (Thermo Fisher cat no 89900) supplemented with protease/phosphatase inhibitors (Thermo Fisher cat no 78440). SDS PAGE was performed using Bio-Rad Criterion TGX gels. Protein was then transferred from the gel onto a nitrocellulose membrane and then the membrane was blocked for 1 hr in 5% non-fat dry milk (NFDM) in TBST at room temperature, incubated at 4°C overnight in the appropriate primary antibody diluted in 5% Bovine Serum Albumin (BSA) in TBST, and washed 4x 5 min in TBST. Membranes were then incubated for 1 hr in the appropriate secondary antibody in 1% NFDM in TBST at room temperature and then washed 4x 5 min in TBST. Primary antibodies included Plin2 (Abcam, ab52356), Plin5 (Novus Biologics, NB110-60509), PPARα (Abcam, ab24509), and 4-HNE (Chemicon, AB5605). Secondary antibodies included anti-rabbit (Jackson, AB_2337913) and anti-goat (Santa Cruz, sc-2056). Membranes were then developed with ECL prime (Cytiva Amersham, RPN2232) and results analyzed using Image J. For H-NE and carbonylation antibodies, the entire lane was analyzed.

### Protein carbonylation level

Heart supernatant homogenized in RIPA buffer was labelled using a Protein Carbonylation Kit (Abcam, cat no ab178020). Levels of protein carbonylation were then assessed via western blot using the antibodies supplied in the Protein Carbonylation Kit. 8 ug protein was loaded for each sample into a 8–16% TGX gel. Western blot was developed and analyzed as described above.

### RT-PCR

RNA from isolated from mouse heart using the Qiagen miRNeasy kit. RNA concentration was determined using Nanodrop and an equal amount of RNA for each sample was added to the cDNA synthesis reaction performed using the iScript cDNA synthesis kit. Roche FastStart Universal SYBR Green Master (Rox) and respective primers were used to assess expression in the generated cDNA samples. Primer sets: FASN, Forward TGCACCTCACAGGCATCAAT Reverse GTCCCACTTGATGTGAGGGG; 36BP4, Forward GGCCCTGCACTCTCGCTTTC Reverse TGCCAGGACGCGCTTGT; SCD1, Forward CGCTGGCACATCAACTTCAC Reverse AAGAACTCAGAAGCCCAAAGC.

### Exercise stress test

Exercise capacity was assessed as described previously [51]. Briefly, mice were acclimated to a mouse treadmill the day before the test. To perform the experiment mice were run on the treadmill at a 10° incline with incremental speed increases (10 min at 10 m/min, 5 min at 12 m/min, 3 min at 15 m/min, and then every 3 min belt speed increased by 1.8m/min until each mouse reached exhaustion). Each mouse was removed from the treadmill upon exhaustion (would no longer run).

### Lipid extraction

Heart tissue was homogenized and then lipids were extracted using the standard Folch method. Lipids were extracted using 2:1 chloroform methanol. After evaporating the chloroform, lipids were redissolved in a 0.5% Triton X-100 diluted in 2-propanol. Samples were stored at -80°C.

### Lipase activity assay

Cardiac lipase activity was measured using a Lipase Activity Assay kit (Cayman Chemical, cat no 700640). Samples were prepared by homogenizing heart tissue in cold PBS with protease and phosphatase inhibitors (Thermo Fisher, cat no 78440) according to kit instructions. The sample were diluted 50-fold in PBS and a total of 20 ug protein was loaded into each sample well of the white 96 well plate. Fluorescence (excitation: 380nm, emission: 510nm) was measured every 30 seconds for 15 minutes at 37’C. For analysis, background fluorescence was accounted for using sample background wells in which the lipase substrate was not added. Lipase activity values were calculated using the thioglycerol standard curve.

### Triglyceride and free fatty acid measurement

TAG was measured in hearts with lipids extracted as described above. Infinity Triglyceride Reagent (Thermo Fisher Scientific cat no TR22421) was used in accordance with manufacturer’s instructions. Serum fatty acid levels were measured in serum using a free fatty acid kit (Wako NEFA-HR(2)). Manufacturer’s instructions were followed. To prepare the serum, the collected blood was left at room temperature for 30 min and then centrifuged at 6000xg for 2 min at 4°C. The serum (supernatant) was stored at -80°C.

### Statistics

T-test or two-way ANOVA with Bonferroni posthoc test were performed as appropriate (p<0.05). Graphpad prism software was used to perform the statistical analyses. Data are presented as Mean±SEM.

## Results

### Cardiac PPARα knockdown does not induce cardiac hypertrophy

**Fig 1:**
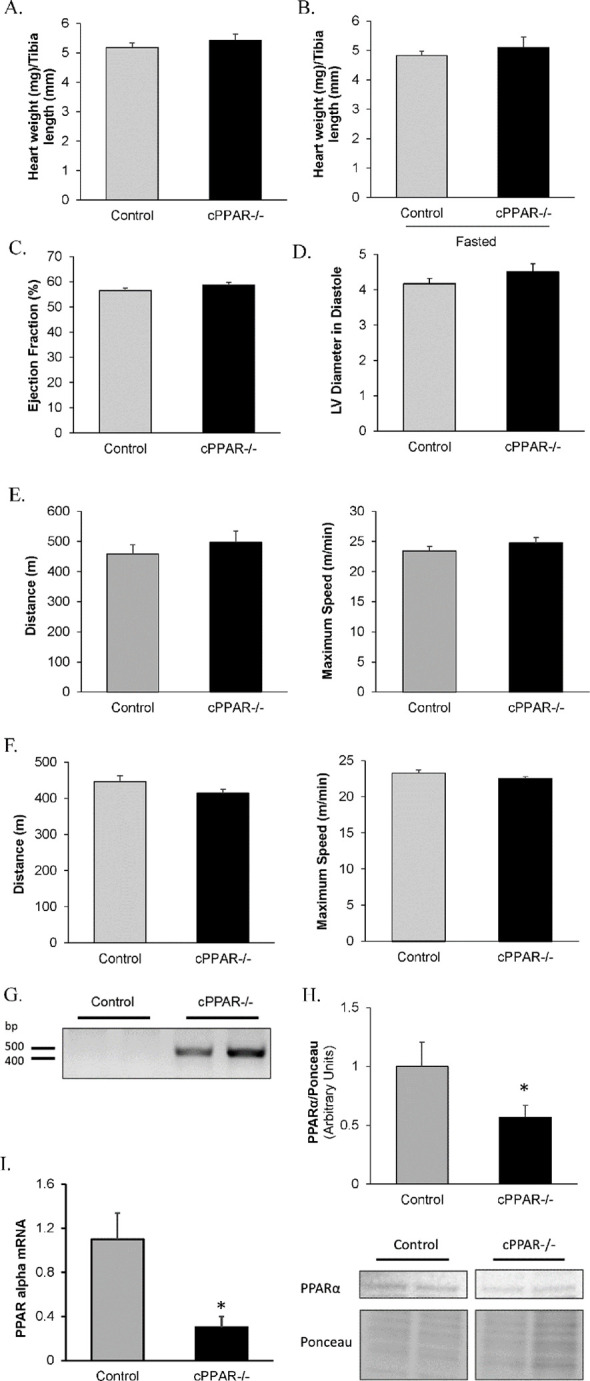
Reduction in cardiac PPARα does not induce cardiac hypertrophy or reduce cardiac function. A. Hearts were weighed at baseline to assess cardiac hypertrophy. n = 12–19. B. Hearts from mice fasted for 16 hrs were weighed. n = 6–10. Heart weights were normalized to tibia length. C. Ejection Fraction and D. LV Diameter in Diastole were measured via echocardiography. n = 6. E. Exercise capacity was assessed via treadmill. n = 5–10. This was done in a single session in one day. F. Exercise capacity was assessed via treadmill in fasted mice. n = 5–6. This was done in a single session in one day. G. Genotyping of mouse tissues using genomic DNA. ~ 450 bp band indicates Mer-Cre-Mer (MCM) cut the loxp flanked sequence in the PPARα gene. H. PPARα protein levels were measured in hearts. n = 6–8. Representative blots are from the same membrane. I. PPARα mRNA levels were measured in hearts. n = 4–5 Values are mean ± SEM. * p<0.05 compared to Control.

PPARα is a key transcriptional regulator of fatty acid metabolism and plays a role in the development of heart disease [2, 8, 21, 22, 30, 32, 44]. Overexpressing PPARα in the heart can induce cardiac dysfunction and cardiac hypertrophy [21–23]. On the other hand, studies have been inconsistent on the effect of whole body PPARα knockout on cardiac function and hypertrophy [24, 30, 42, 44, 52]. To better understand the impact of myocardial PPARα, we generated an inducible cardiac-specific PPARα knock-out (cPPAR^-/-^) mouse. We observed no evidence of cardiac hypertrophy in cPPAR^-/-^ vs control mice (Fig 1A, 1B and 1D) at baseline. Heart weight/tibia length was also similar in hearts from fasted Control and cPPAR-/- mice (Fig 1B). These measurements were made in 3–4 month old mice after waiting 1–2 months after tamoxifen injection. Further, cPPAR^-/-^ mice have a similar cardiac function and exercise capacity to control mice (Fig 1C and 1E). After the 16 hr fast, exercise capacity was similar between cPPAR-/- and control mice (Fig 1F). Genotyping, mRNA, and protein all indicated a reduction in PPARα in hearts after tamoxifen treatment (Fig 1G–1I).

### Cardiac PPARα knockdown reduces lipid accumulation during fasting despite similar fatty acid supply

**Fig 2:**
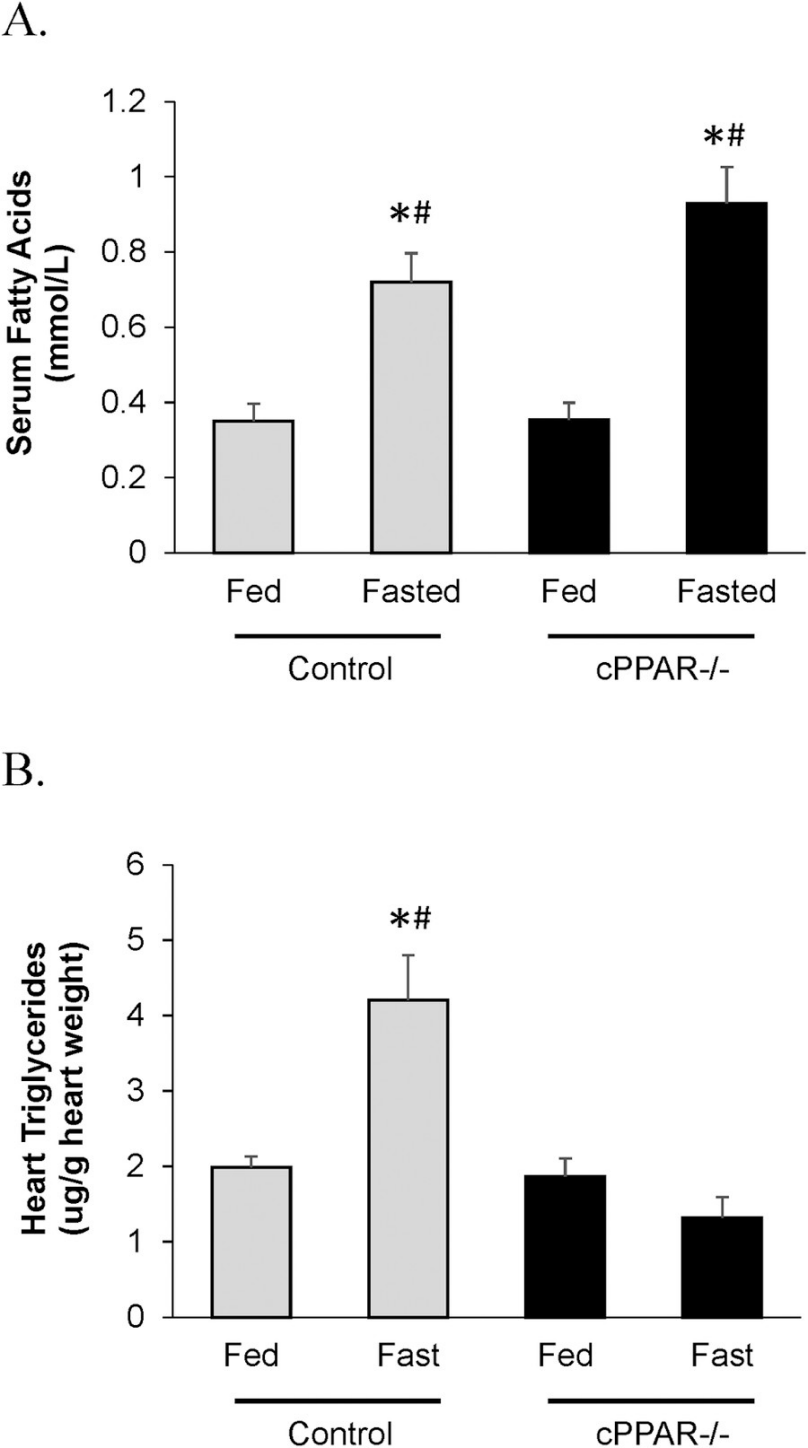
Reduction in myocardial PPARα prevents cardiac triglyceride accumulation. Mice were fasted for 16 hrs and then A. serum fatty acid levels and B. triglyceride levels were measured in hearts. Values are mean ± SEM; n = 5–12; * p<0.05 compared to Control Fed, ^#^ p<0.05 compared to cPPAR^-/-^ Fed.

One of the ways PPARα has been linked to cardiac dysfunction and hypertrophy is through regulation of lipid accumulation. However, since both whole body PPARα knockout and cardiac PPARα overexpression increases induction of lipid accumulation in the heart [2, 41–43, 53], it is unclear whether a reduction in cardiac PPARα drives or protects against lipid accumulation in the heart. As mentioned in the whole body PPARα knockout mouse there are systemic effects that can complicate the interpretation. We utilized the cPPAR^-/-^ mice to test whether a reduction in cardiac PPARα impacts the response of the heart to an acute elevation in fat supply. Circulating fatty acids and cardiac TAG levels become elevated in response to a short term fast. As expected, the level of circulating fatty acids in response to a 16 hr fast was similar between cPPAR^-/-^ and control mice (Fig 2A). Thus, it was perhaps surprising that in response to fasting, and an elevation in circulating fatty acid levels, cPPAR^-/-^ hearts had dramatically lower cardiac TAG levels than control mice (Fig 2B). This indicates that a reduction in cardiac PPARα attenuates cardiac lipid accumulation, at least under a short term elevation in fatty acid supply to the heart.

### Cardiac specific PPARα knockout mouse hearts have reduced PLIN2

**Fig 3:**
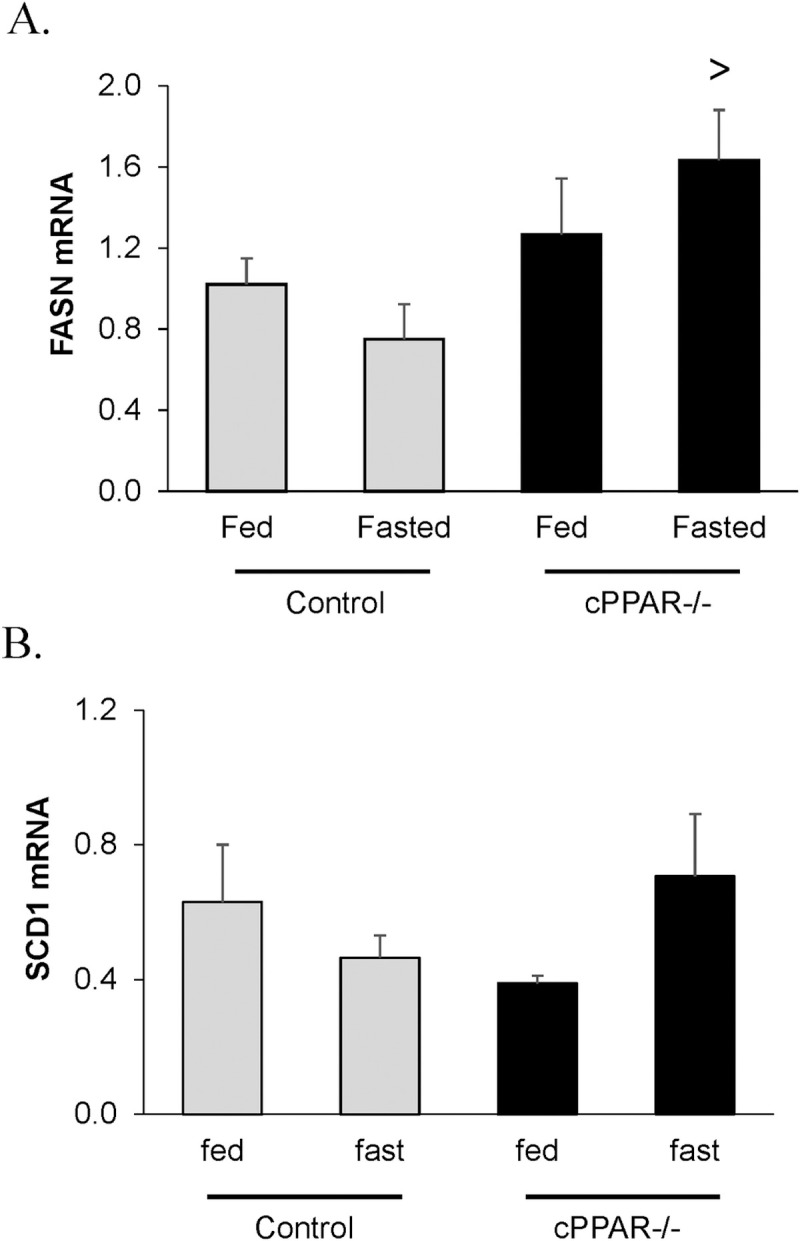
FASN was not altered in fasting in mice with reduced myocardial PPARα. Mice were fasted for 16 hrs and then A. FASN mRNA and B. SCD1 was measured in the hearts. Values are mean ± SEM. n = 5–11; ^>^ p<0.05 compared to Control Fasted.

We therefore were interested in understanding how a reduction in cardiac PPARα reduces accumulation of TAGs in the heart. We hypothesized that the mechanism might involve alterations in cardiac accumulation or breakdown of TAG stores. Since PPARα has been linked to regulating SREBP [54], a transcriptional regulator of fatty acid synthesis, we first examined whether there might be decreased expression of genes regulated by SREBP1c. Interestingly, we did not observe lower fatty acid synthase (FASN) mRNA under baseline or fasting conditions between cPPAR^-/-^ and control hearts (Fig 3A). The elevation in FASN mRNA in fasted cPPAR^-/-^ vs Control hearts may be a compensatory increase in response to overall reduced TAG synthesis in the fasted cPPAR^-/-^ hearts.

**Fig 4:**
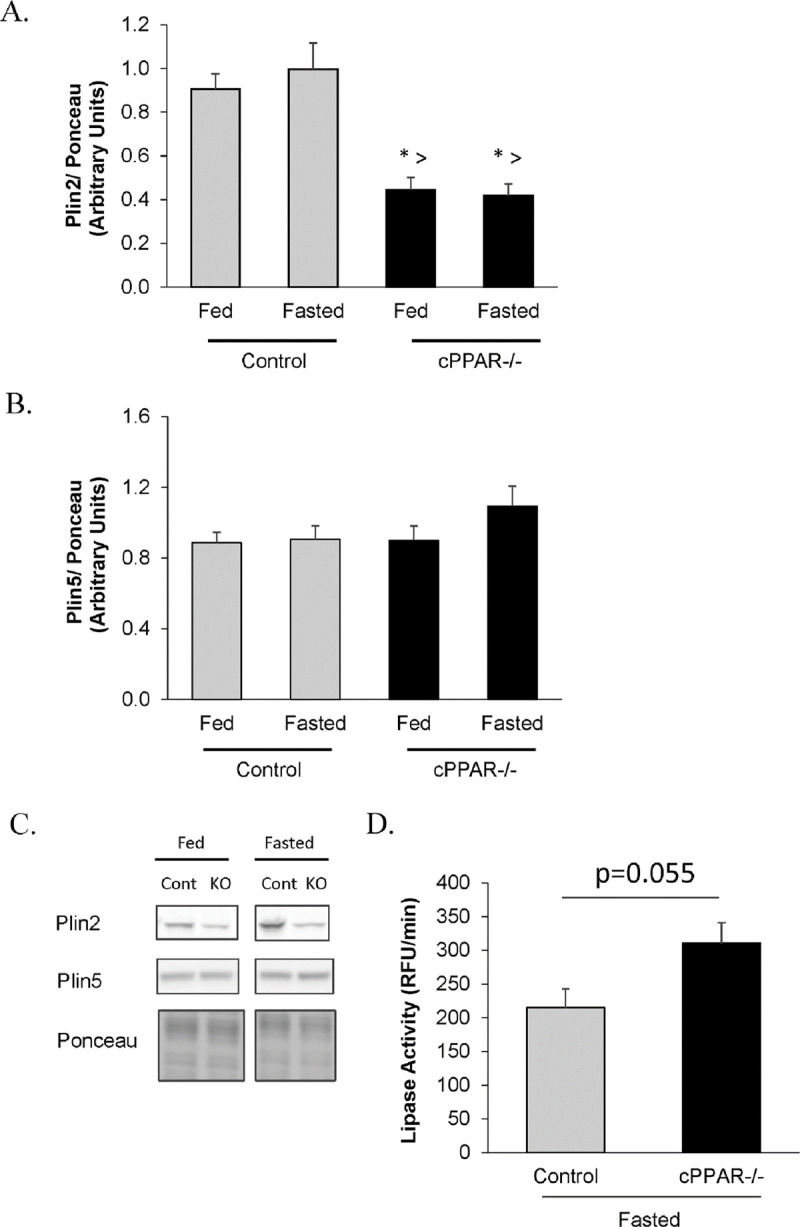
Reduction in myocardial PPARα mice lowers cardiac Plin2. Mice were fasted for 16 hrs and A. Plin2 and B. Plin5 protein levels were measured in heart. C Representative western blots [Cont (Control); KO (cPPAR-/-)]. D. Lipase activity in heart. Values are mean ± SEM; n = 5–12; * p<0.05 compared to Control Fed; ^>^ p<0.05 compared to Control Fasted.

Recently, it has become recognized that Plin also regulates the accumulation of TAG [19, 55], and that a loss of Plin2 can lead to reduced lipid accumulation. Plin2, in particular, is believed to play an important role in controlling lipid droplet and triglyceride accumulation [17–20, 56]. We, therefore, examined whether Plin2, a Plin that is highly expressed in the heart, was altered in the cPPAR^-/-^ hearts. Interestingly, cardiac Plin2 was lower at baseline cPPAR^-/-^ vs control hearts. Expression was also lower under fasting conditions in cPPAR^-/-^ hearts (Fig 4A). This difference in Plin was Plin2 specific since Plin5 levels were not different between cPPAR^-/-^ and control hearts (Fig 4B).

### Cardiac specific PPARα knockout mouse hearts do not show evidence of increased protein oxidation

**Fig 5:**
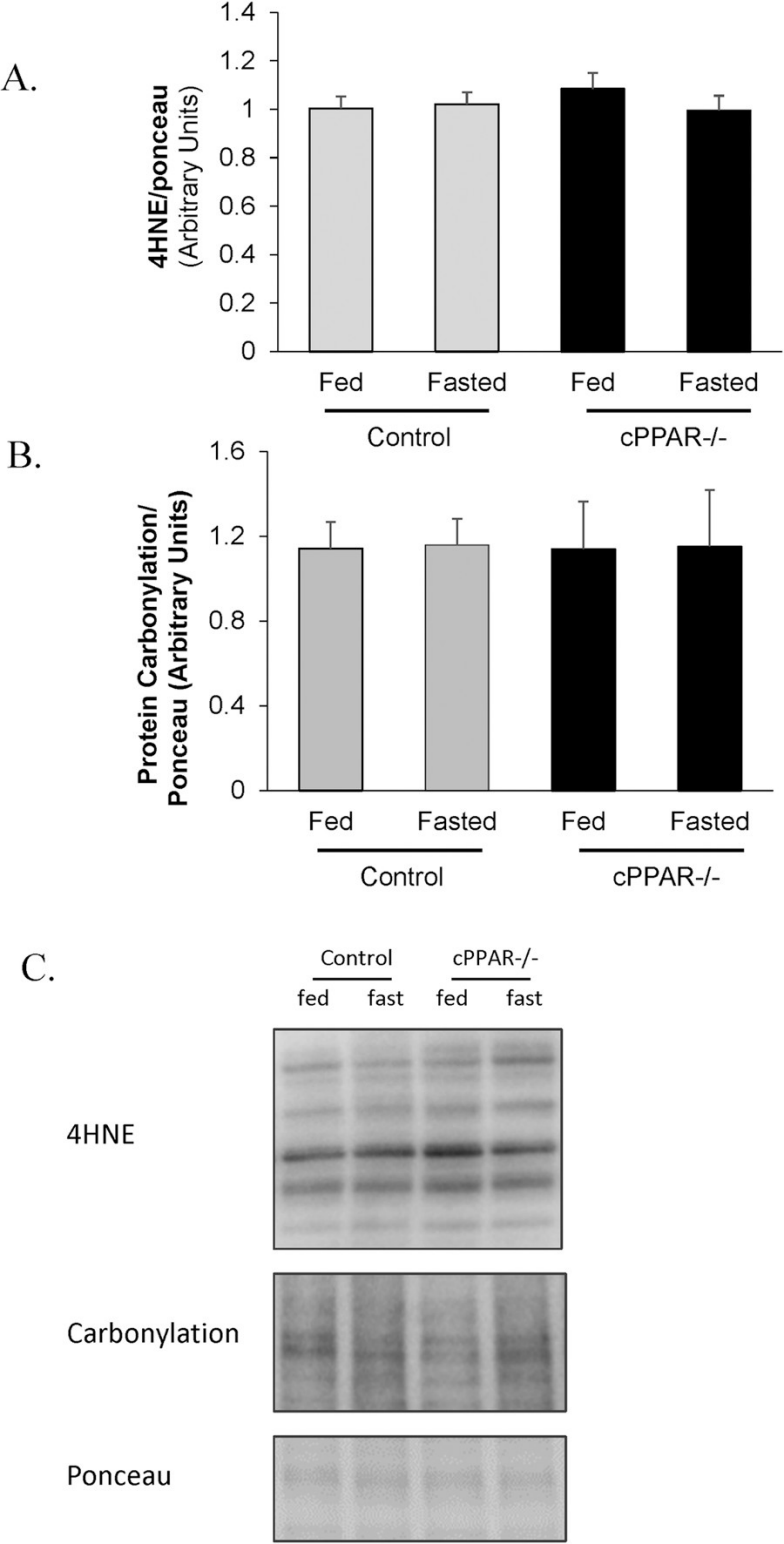
Control or fasted mice with reduced myocardial PPARα do not have elevated levels of protein oxidation. Mice were fasted were 16 hrs and then A. 4-HNE and B. protein carbonylation levels were measured in heart. C. Representative western blot images. Values are mean ± SEM. n = 5–12.

Lipid accumulation can have detrimental effects through increasing oxidative injury. We considered the possibility that the attenuated TAG accumulation in the cPPAR^-/-^ hearts might be associated with accumulation of other lipid species that might lead to lipid peroxidation and protein oxidation which can have detrimental effects in the heart. To check this, we first measured 4-HNE as an indicator of lipid peroxidation. Importantly, there was no evidence of 4-HNE levels being elevated in cPPAR^-/-^ hearts (Fig 5A). Further, protein carbonylation was also not elevated in cPPAR^-/-^ hearts (Fig 5B). This was the case under both control and fasted conditions. This indicates that oxidation and peroxidation were not elevated in cPPAR^-/-^ hearts.

## Discussion

A role for the transcription factor PPARα in fatty acid metabolism, cardiac function, and hypertrophy was reported more than two decades ago [2, 8, 21, 22, 30, 31, 33, 37, 38, 44, 57]. In this study, we demonstrate for the first time the effect of a cardiac specific decrease in PPARα *in vivo*. While it is clear that PPARα regulates fatty acid metabolism it is unclear whether PPARα reduces or exacerbates cardiac lipid accumulation.

Our aim in the current study was to examine the role of myocardial PPARα in regulating TAG accumulation. We fasted the mice to increase adipocyte lipolysis, thereby increasing the supply of fat to the heart; this normally results in a dramatic increase in the amount of TAG accumulation in the heart. Because whole body PPARα knockout mice showed a much larger increase in cardiac lipid levels under conditions that stimulate cardiac lipid accumulation [42, 43], we expected that fasting would also induce an increase in lipid accumulation in the cPPAR^-/-^ compared to control hearts. In contrast to our hypothesis, cPPAR^-/-^ hearts exhibited less cardiac lipid accumulation (Fig 2). TAG levels were ~3 fold lower in fasted cPPAR^-/-^ hearts vs fasted control hearts. This occurred even though there was a similar level of circulating fatty acids in fasted cPPAR^-/-^ and control mice (Fig 2).

One possible explanation for the reduction in TAG accumulation could be less fatty acid synthesis. In particular, we examined whether targets of SREBP (which participate in fatty acid synthesis) might be reduced. SREBP1c was examined because it regulates the expression of proteins involved in fatty acid synthesis and triglyceride accumulation [58] While the literature did not cause us to expect FASN to increase in response to fasting in Control mouse hearts, a lower expression of FASN in cPPAR^-/-^ vs Control hearts might help explain the difference in TG accumulation we observed [59]. However, the expression of FASN was not lower in cPPAR^-/-^ compared to control hearts (Fig 3). It is still possible, however, that there was a change in the expression and/or activity of proteins involved in TAG synthesis. We also investigated a role for Plin2, as it has been reported to regulate TAG levels. We did not observe a difference in Plin2 levels in fed vs fasted hearts which agrees with Varghese et al. who do not see a total change in Plin2 in heart homogenate but instead an increase in Plin2 localization to lipid droplets [14]. Plin2 protein was lower in cPPAR^-/-^ vs control in fasted hearts (Fig 4A) suggesting it could be involved in the reduced lipid levels in cPPAR^-/-^ fasted hearts. These data are consistent with the hypothesis that reduced levels of cardiac PPARα during short term fasting prevents cardiac lipid accumulation due to regulation of lipid storage, potentially because of increased activity of lipases such as ATGL.

Plin2 is a member of a family of proteins that bind to the outside of LD. LD store neutral lipids including TAG within a phospholipid membrane. Plin have been shown to regulate LD turnover through multiple mechanisms. Plin can both shield LD from lipases and regulate the activity of lipases ATGL and HSL on lipid droplets [14–20]. Mice with cardiac specific overexpression of Plin2 [17] developed cardiac steatosis suggesting that Plin2 opposes breakdown of LD. In this study, a moderate reduction in cardiac Plin2 is associated with a reduction in cardiac lipid levels. An important question for understanding fatty acid metabolism and its role in disease is how Plin and lipid droplets are regulated. Our study and others suggest that PPAR is an important regulator of Plin [47–50, 60, 61]. In particular, the data in our study links a reduction in cardiac PPARα to a reduction in cardiac Plin2, indicating that cardiac PPARα is an important regulator of cardiac Plin2 expression.

Changes in Plin have been linked to changes in oxidative stress and cardiac dysfunction [18, 56, 62, 63]. In particular, a rise in the lipid species diacylglycerol (DAG) and ceramides have been linked to cardiac lipotoxicity [64–67]. Cardiac DAG increases along with TAG in mice with cardiac Plin2 overexpression [17]. Interestingly, studies have shown that increasing flux toward TAG and away from DAG and ceramides seems to reduce this lipotoxicity [56, 65, 66]. We therefore wanted to make sure the reduced TAG in fasted cPPAR^-/-^ hearts was not associated with increased oxidative stress. Notably, levels of oxidative stress were similar between fasted cPPAR^-/-^ hearts and control hearts. This also indicates that the change in Plin2 does not appear to be associated with the deleterious results that have been reported in some models where lipid storage is dysregulated [18, 56, 62, 63]. It will be interesting in the future to investigate how specific lipids (including DAGs and ceramides) are different between cPPAR^-/-^ hearts and control hearts. Further studies will provide novel insight into the regulation of lipid species and how specific lipid species change in lipid droplets. This indicates that at least under physiological conditions, a decline in PPARα decreases cardiac Plin2 without inducing protein oxidation or cardiac hypertrophy. Further studies will be needed to investigate the direct link between this protection against cardiac triglyceride accumulation and the development of cardiac hypertrophy using disease models.

## Conclusions

In summary, this study finds that a reduction in cardiac PPARα has a dramatic impact on cardiac lipid accumulation. A 16 hr fast resulted in less cardiac lipid accumulation in cPPAR^-/-^ compared to control hearts. This was accompanied by lower levels of cardiac Plin2 protein. Furthermore, cPPAR^-/-^ hearts did not have higher markers of protein oxidation or cardiac hypertrophy when compared to control. Overall, these data suggest that decreased cardiac PPARα reduces cardiac lipid accumulation in the heart, and could potentially protect against diseases with dysregulated lipid storage such as diabetic cardiomyopathy.

## Supporting information

S1 Raw images (PDF)
