# Supplementary material for: Cardiac specific knock-down of peroxisome proliferator activated receptor α prevents fasting-induced cardiac lipid accumulation and reduces perilipin 2
Source: PLoS One. 2022 Mar 8;17(3):e0265007. doi: 10.1371/journal.pone.0265007 (PMC8903264; doi:10.1371/journal.pone.0265007)
Supplement: S1 Raw images — (PDF) [file pone.0265007.s001.pdf]

Legend- Control indicated as: C
cPPAR-/- indicated as: KO

From Fig 1G

bp ladder

X C C C C C KO C C C

bp

500
400
300
200
100

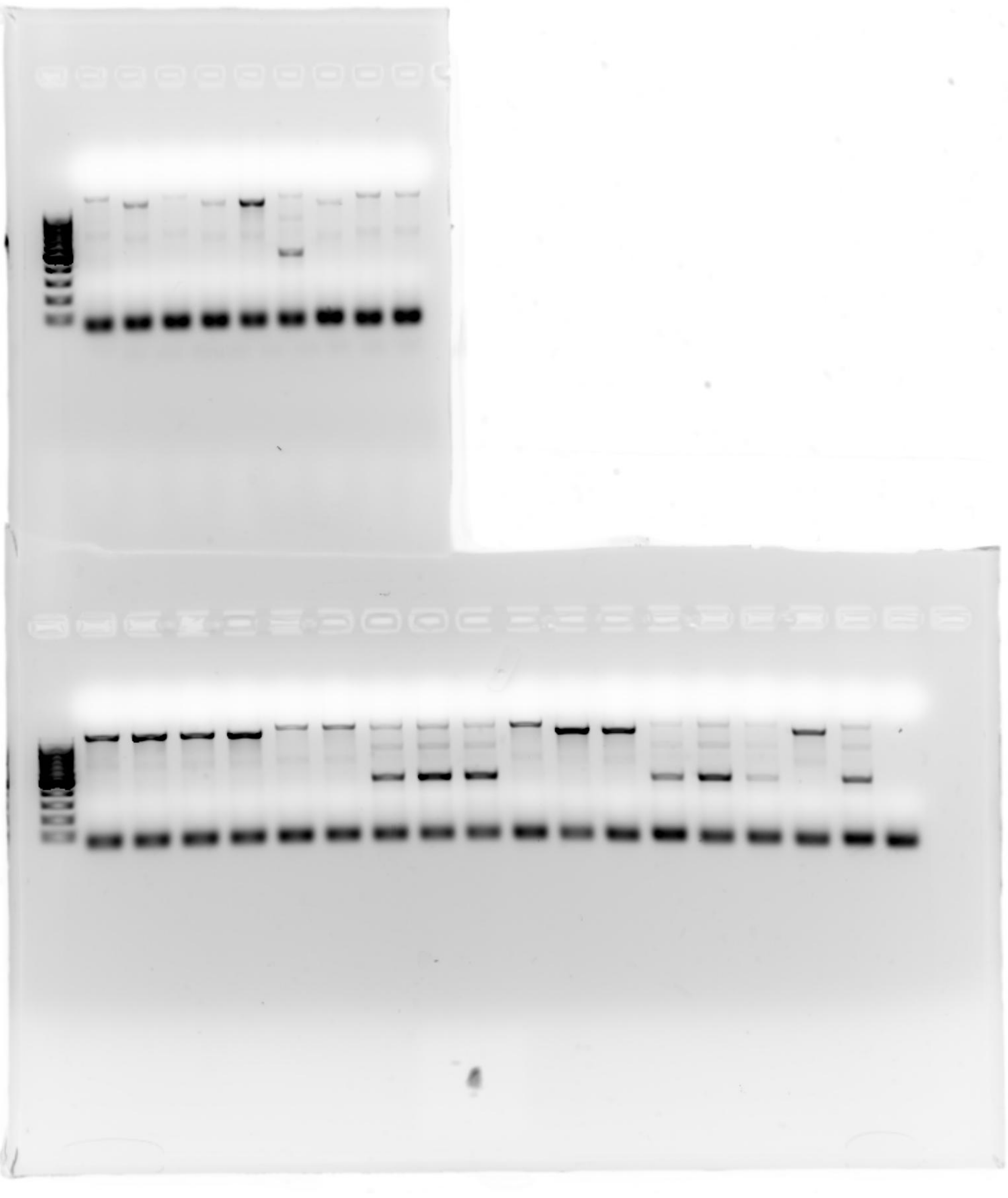

bp

500
400
300
200
100

X C C C C C C KO KO KO C C C KO KO KO C KO C

From Fig 1H

Legend- Control indicated as: C
cPPAR-/- indicated as: KO

PPAR alpha

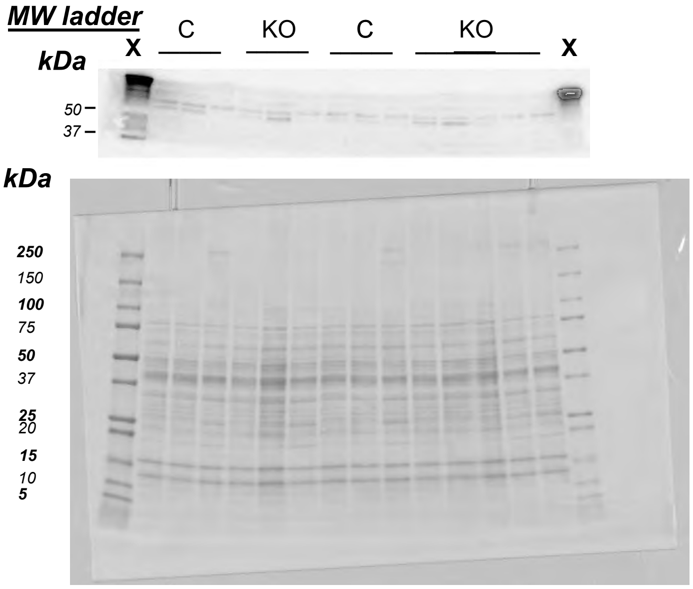

Ponceau

From Fig 4C

Legend- Control indicated as: C

cPPAR-/- indicated as: KO

F: indicates the mouse was fasted. If there is no F that means the sample was from a mouse fed ad libitum.

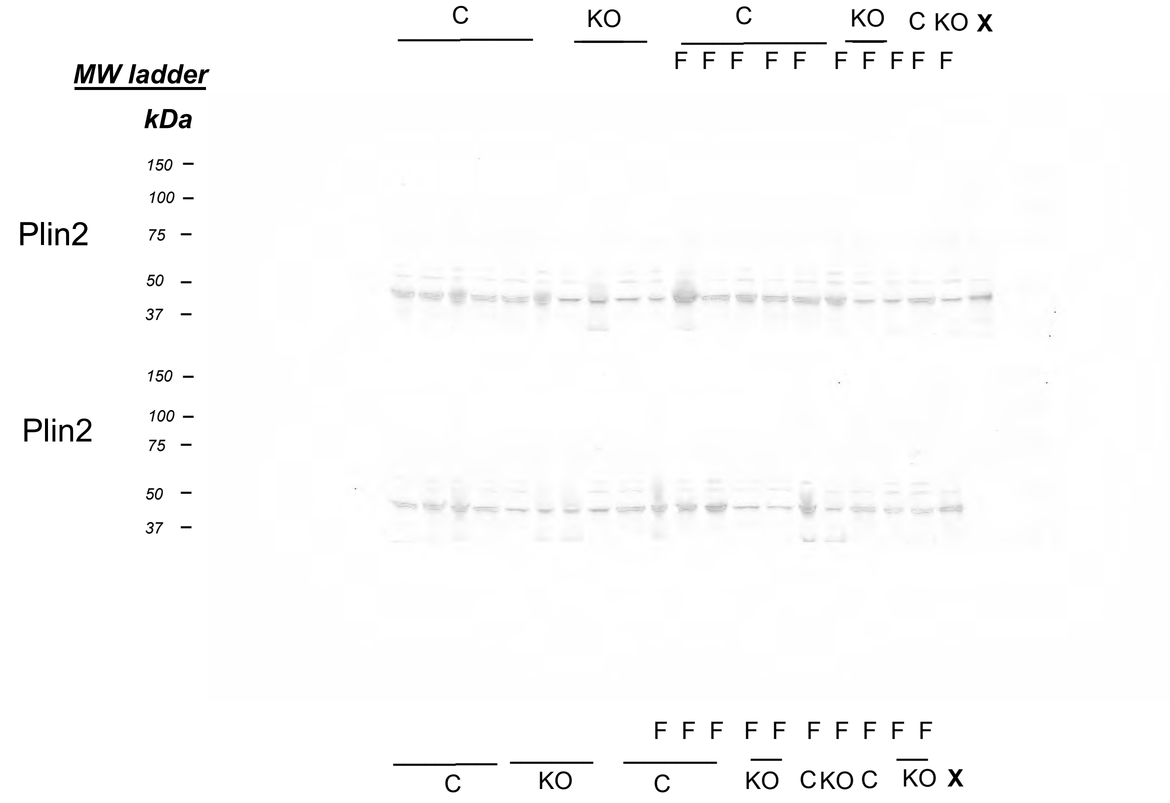

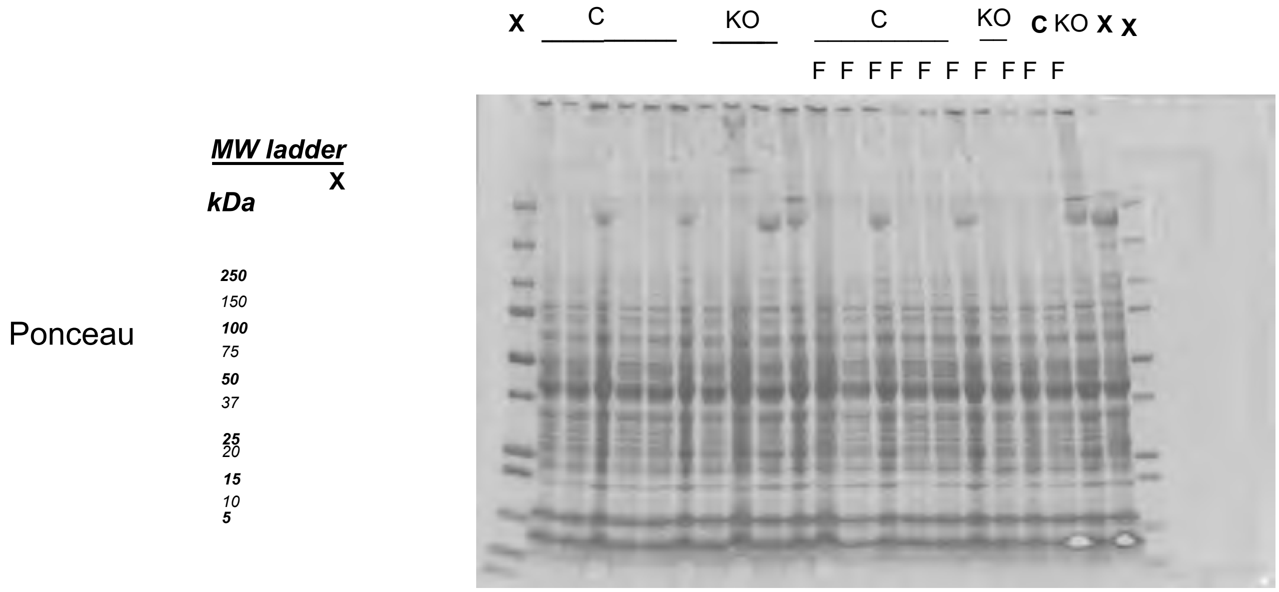

X C KO C KO C KO C KO X X X

F F F F F F F F F F

MW ladder

X

kDa

Ponceau

250
150
100
75
50
37
25
20
15
10
5

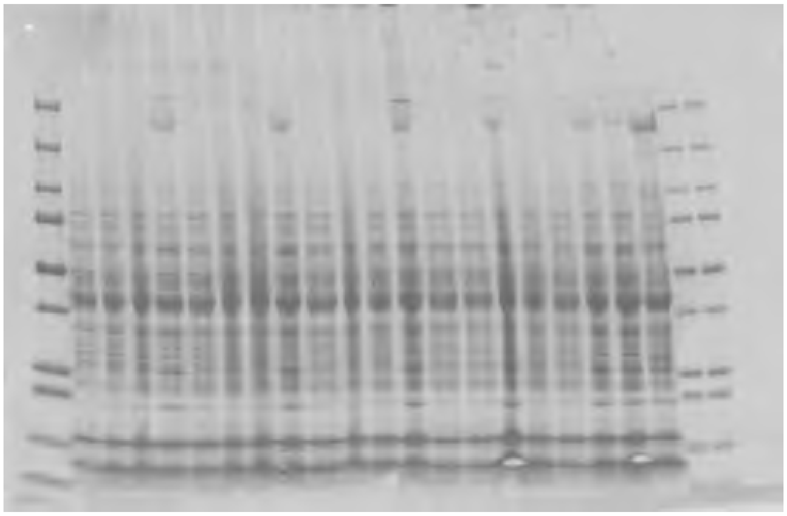

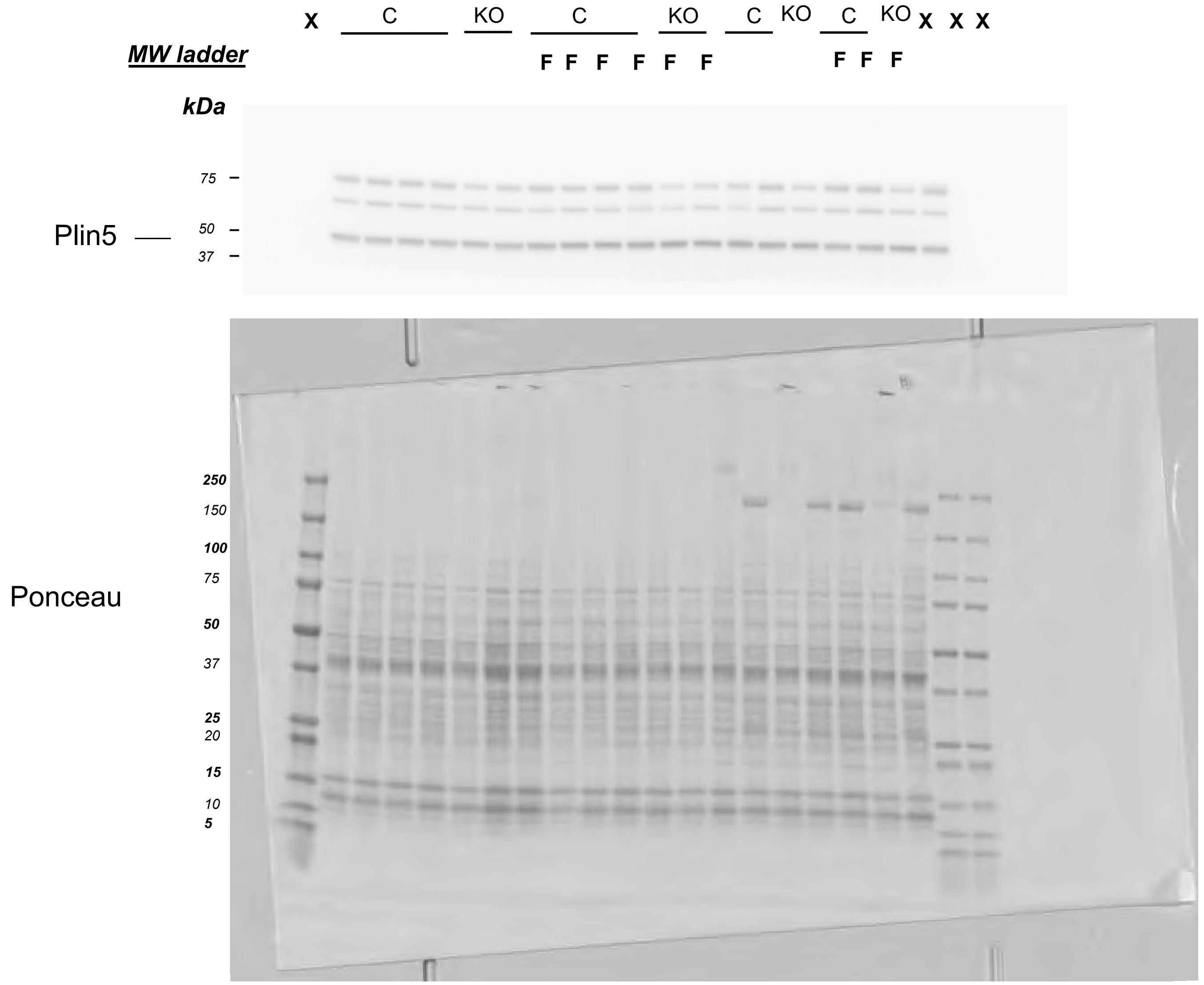

X
C
KO
C
KO
C
KO
C
KO
X X X
MW ladder
F F F F F F
F F F
kDa
75
50
37
Plin5
Ponceau
250
150
100
75
50
37
25
20
15
10
5

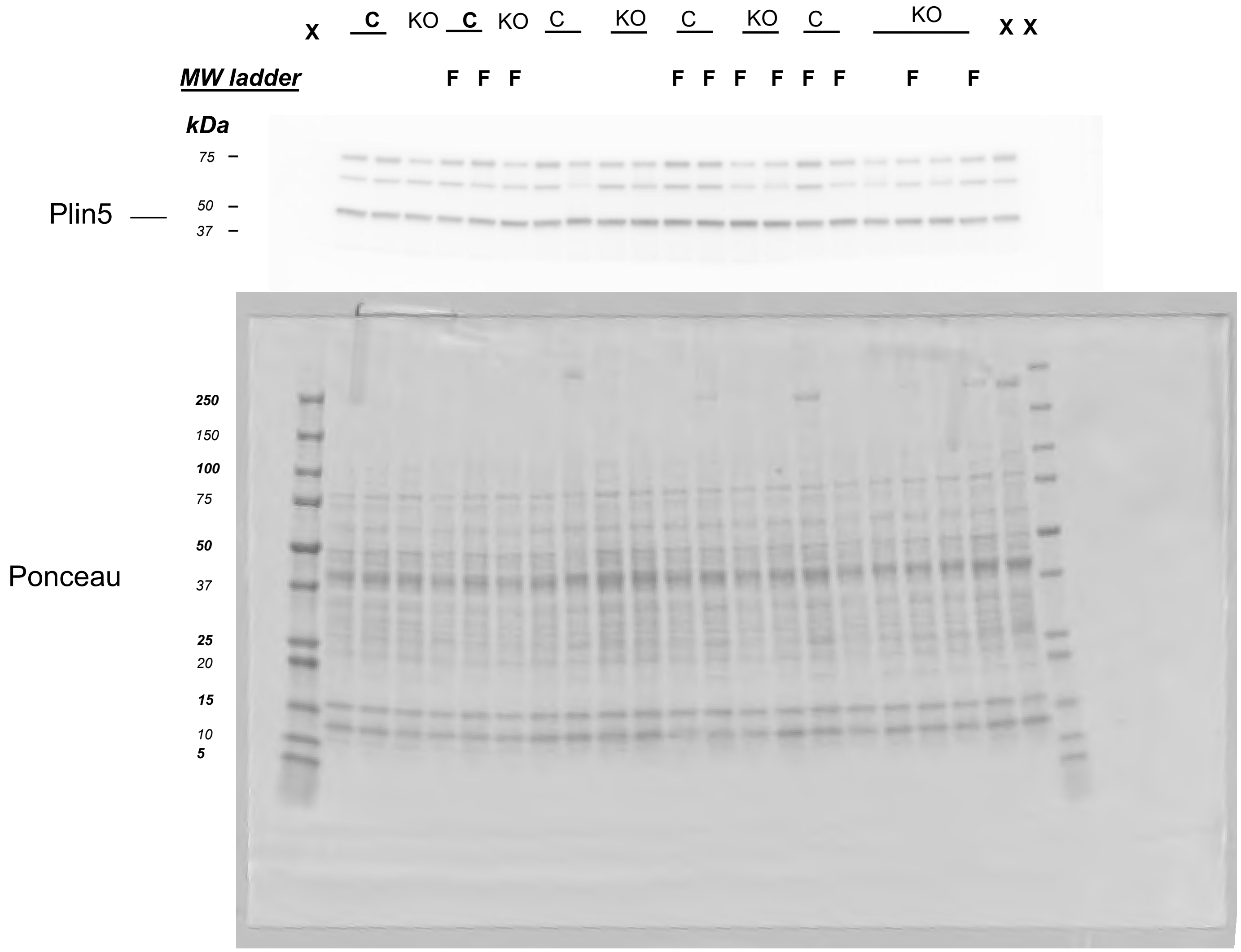
X
C
KO
C
KO
C
KO
C
KO
C
KO
X X
MW ladder
F F F F F F F F F F F
kDa
75
50
37
Plin5
Ponceau
250
150
100
75
50
37
25
20
15
10
5

Legend- Control indicated as: C

cPPAR-/- indicated as: KO

F: indicates the mouse was fasted. If there is no F that means the sample was from a mouse fed ad libitum.

From Fig 5C

X C KO C KO C KO **C** KO C KO **C** KO **X**

MW ladder F F F **F** F F F F F

kDa

250
150
100
75
50
37
25
20
15
10
5

4HNE

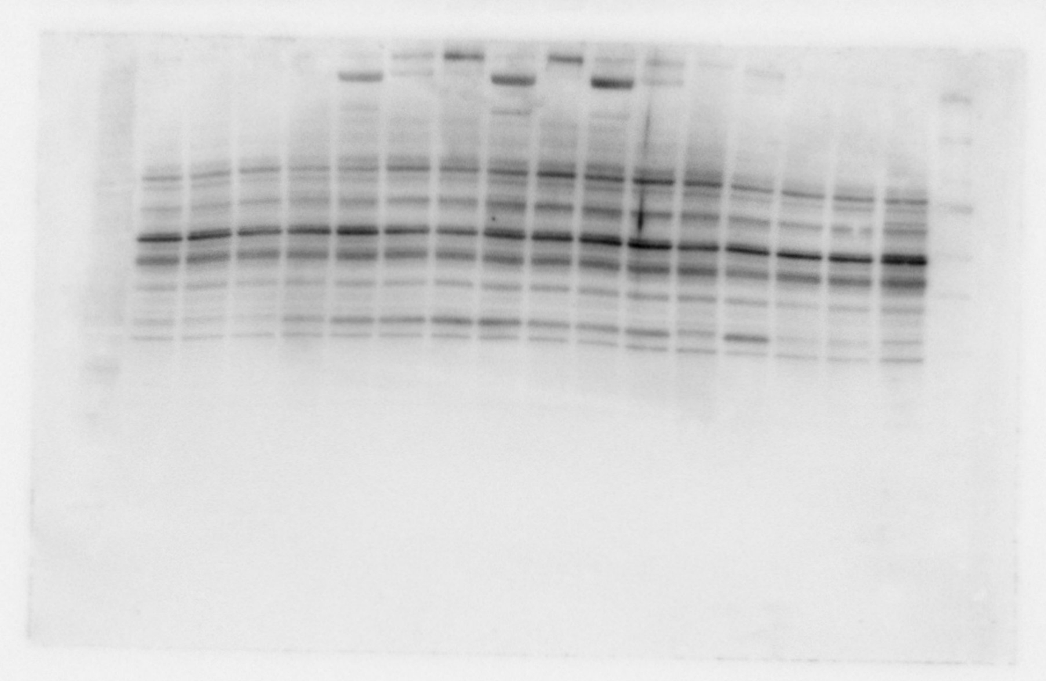

250
150
100
75
50
37
25
20
15
10
5

Ponceau

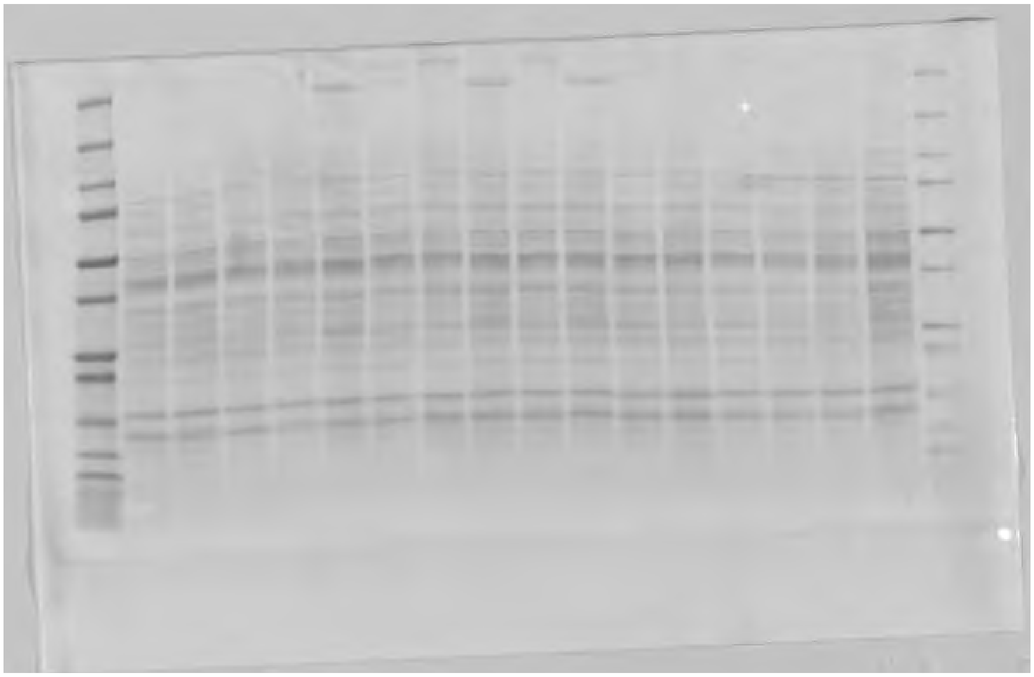

X C KO C KO C KO C KO C KO C C KO
F F F F F F F F F

MW ladder
kDa

4HNE

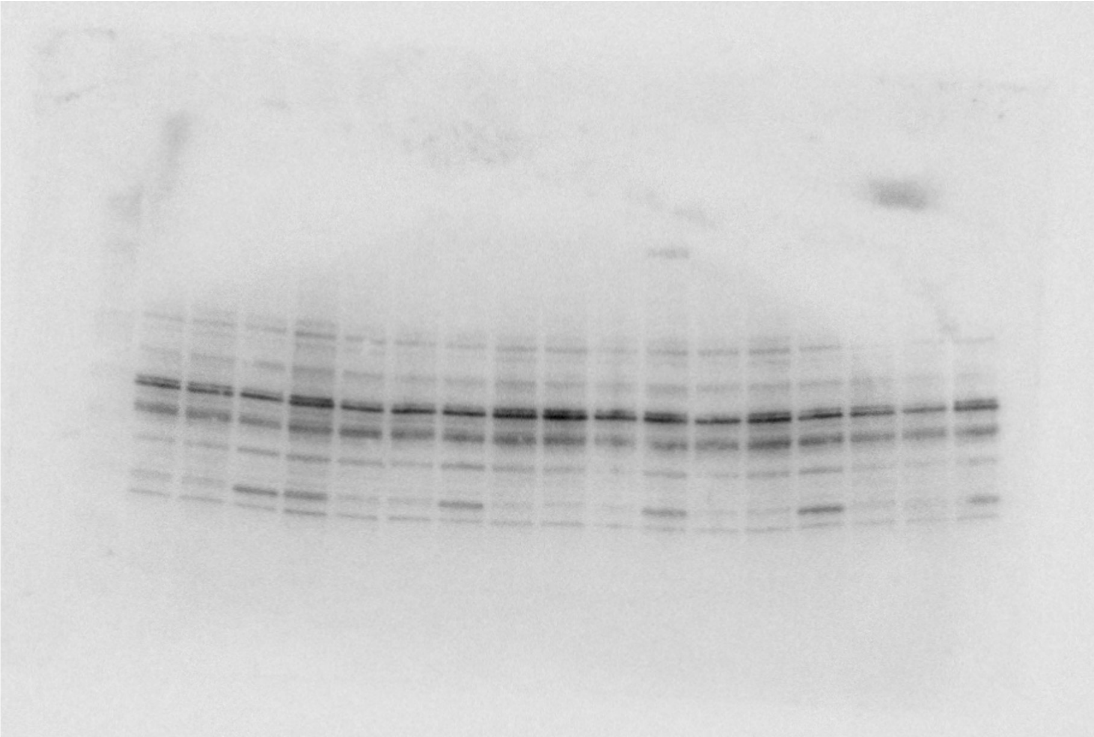

Ponceau

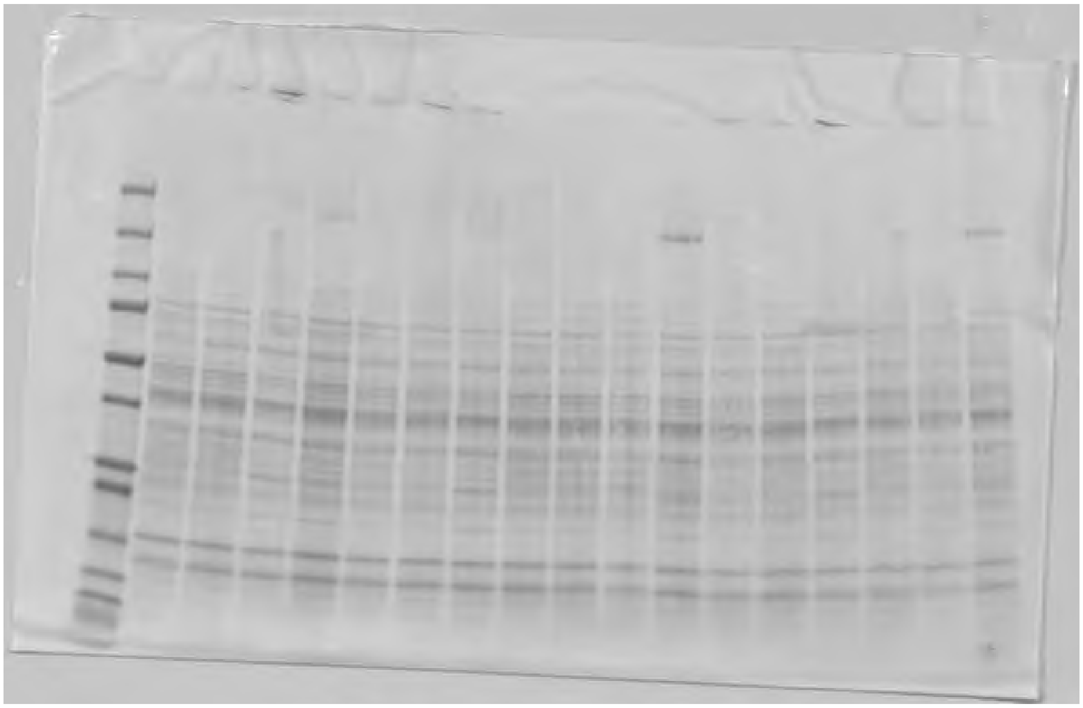

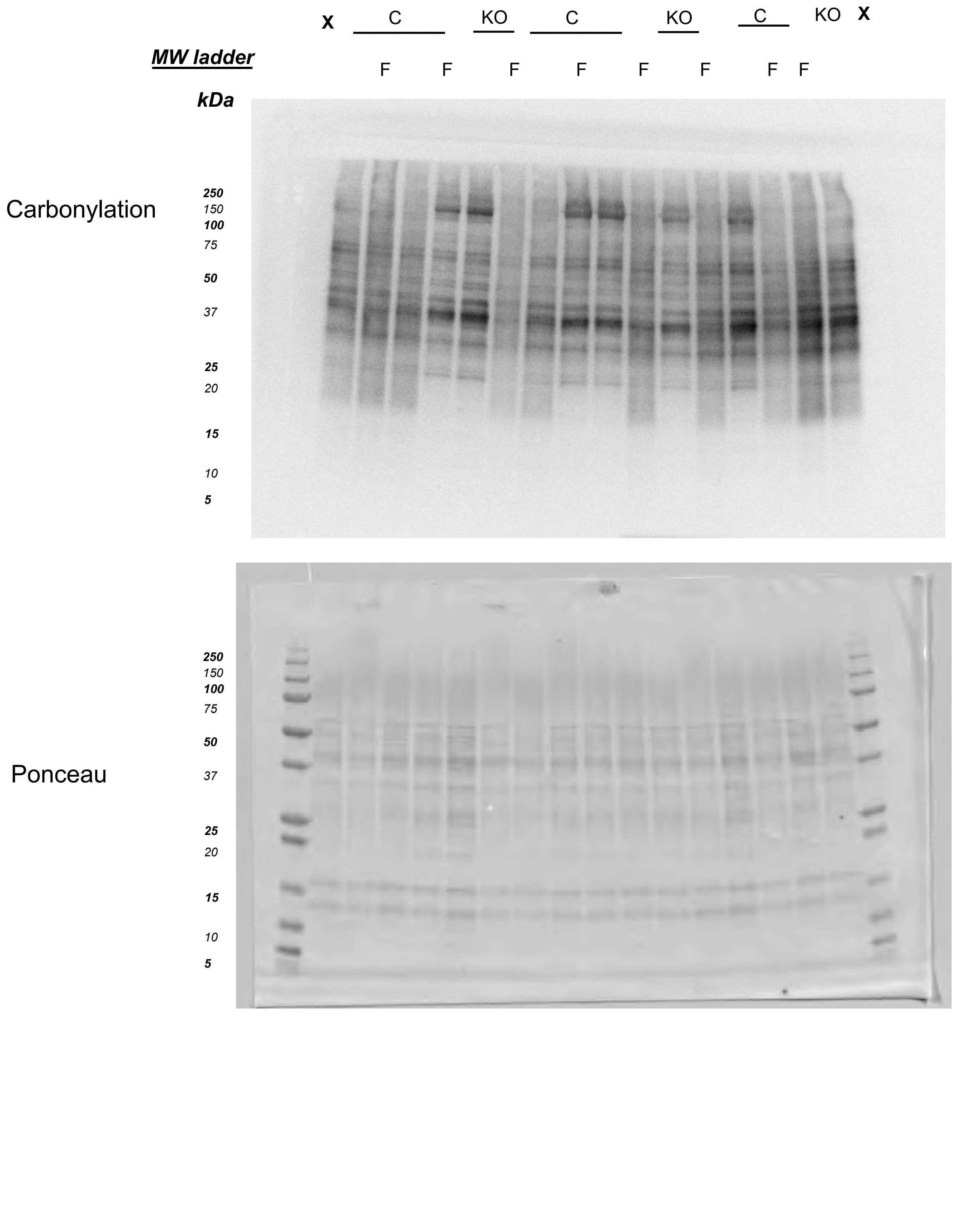
X
C
KO
C
KO
C
KO
X
MW ladder
F F F F F F F F
kDa
Carbonylation
250
150
100
75
50
37
25
20
15
10
5
Ponceau
250
150
100
75
50
37
25
20
15
10
5

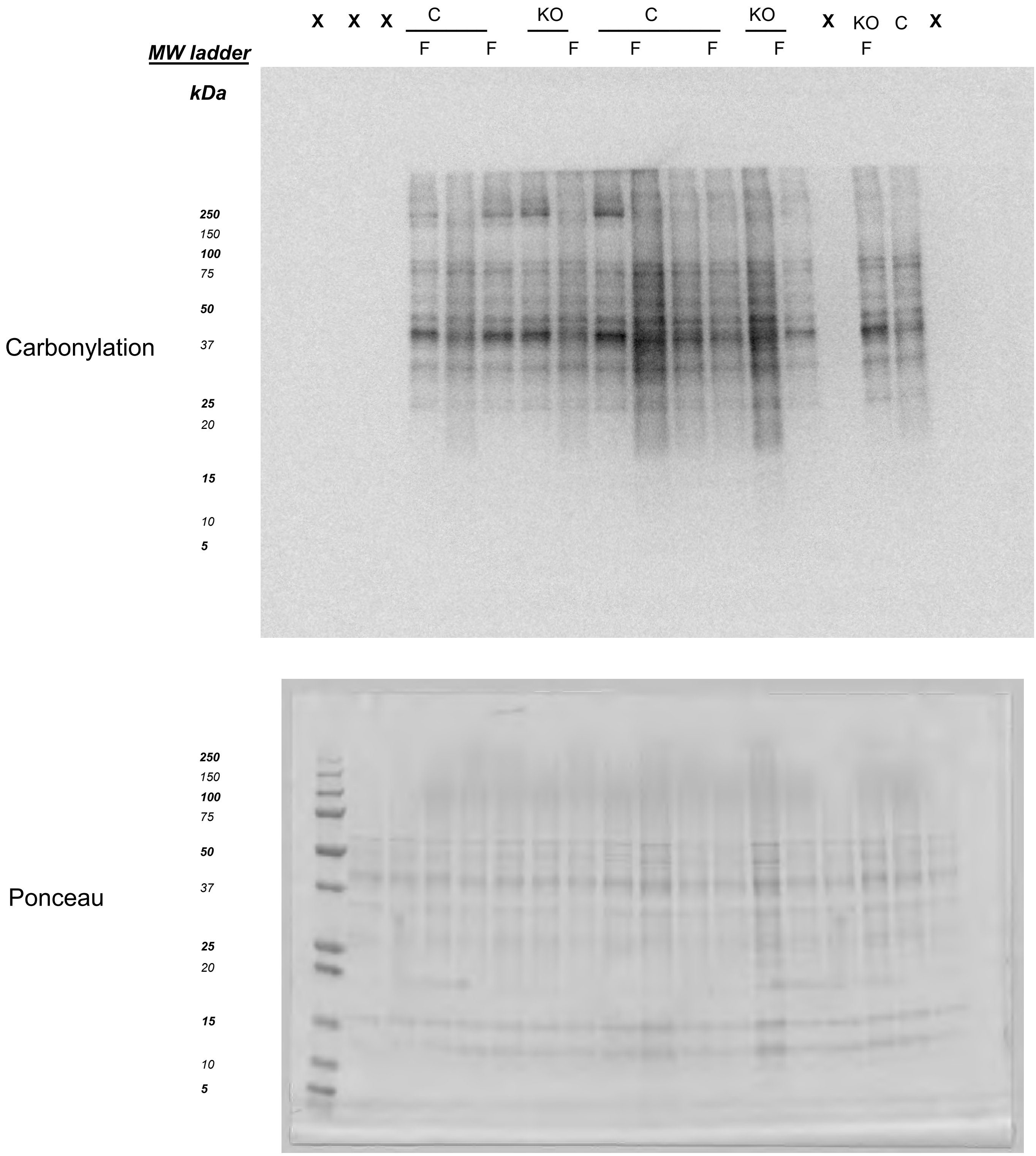

X X X C KO C KO X KO C X
F F F F F F F
MW ladder
kDa
250
150
100
75
50
37
25
20
15
10
5
Carbonylation
Ponceau
